# Case Report: Sorafenib-induced rhabdomyolysis in a post-transplant patient with acute myeloid leukemia

**DOI:** 10.3389/fonc.2026.1728530

**Published:** 2026-02-17

**Authors:** Liuxian Ke, Lingjuan Cao, Shang Li, Zijian Zhou, Ping Zheng

**Affiliations:** 1 Department of Pharmacy, Nanfang Hospital, Southern Medical University, Guangzhou, China; 2 Department of Pharmacy, Guangdong Province Work Injury Rehabilitation Hospital, Guangzhou, China

**Keywords:** acute myeloid leukemia, case report, hypokalemia, rhabdomyolysis, sorafenib

## Abstract

**Background:**

Rhabdomyolysis is a clinical acute syndrome characterized by acute injury or necrosis of skeletal muscle cells and may be life-threatening. We report a rare case of rhabdomyolysis caused by sorafenib.

**Case:**

This case report presents a 31-year-old female patient who experienced rhabdomyolysis during sorafenib treatment following allogeneic hematopoietic stem cell transplantation. The patient underwent the transplantation due to acute myeloid leukemia (AML) and subsequently received sorafenib as maintenance therapy to prevent leukemia relapse. During treatment, she developed bilateral lower limb weakness, a significant increase in creatine kinase (CK) levels, and hypokalemia. After discontinuing sorafenib and initiating supportive treatments, her CK levels gradually decreased. Neuromuscular biopsy results showed rhabdomyolysis and immune-mediated necrotizing myopathy.The patient was followed up in the outpatient clinic after discharge, and the symptoms of myasthenia in both lower limbs have improved. On the 38th day after discharge, laboratory tests showed that the levels of α-hydroxybutyrate dehydrogenase, creatine kinase, lactate dehydrogenase, and electrolytes had returned to normal.

**Conclusion:**

This case highlights that sorafenib may cause rare but severe adverse reactions, such as rhabdomyolysis. Clinicians should be vigilant about these potential adverse effects when using sorafenib and enhance patient monitoring and management.

## 1 Introduction

Acute myeloid leukemia (AML) is a cancer of myeloid progenitor cells, with patients harboring FLT3 mutations typically classified as high-risk. Allogeneic hematopoietic cell transplantation (HCT) can significantly improve the long-term survival rate of patients with FLT3 mutations. Following transplantation, patients may continue to receive FLT3 inhibitors for maintenance therapy to prevent leukemia relapse (1).

Sorafenib is an oral multi-targeted tyrosine kinase inhibitor that can inhibit various kinases, including BRAF, RET, KIT, VEGFR, Ras/Raf, and PDGF, etc. (2). In addition to targeting Raf serine/threonine kinases, sorafenib also potently inhibits the proangiogenic vascular endothelial growth factor receptor (VEGFR)-1, VEGFR-2, VEGFR-3, and platelet-derived growth factor receptor-β (PDGFR-β) tyrosine kinases in biochemical assays *in vitro*. In cellular assays, sorafenib inhibits the VEGF-mediated autophosphorylation of VEGFR-2 (human endothelial cells and NIH 3T3 fibroblasts expressing VEGFR-2), VEGFR-3, and PDGF-mediated autophosphorylation of PDGFR-β in HAoSMCs (3). It is FDA-approved for the treatment of unresectable hepatocellular carcinoma (HCC) and advanced renal cell carcinoma (RCC) patients (4). Clinical studies of sorafenib for maintenance therapy in FMS-like tyrosine kinase 3 gene (FLT3-ITD) internal tandem duplication mutation acute myeloid leukemia (AML) have shown that it can reduce the recurrence and death risk of FLT3-ITD internal tandem duplication mutation AML after allogeneic hematopoietic stem cell transplantation (HCT) (5). Sorafenib mainly targets FLT3 mutations, especially FLT3-ITD (internal tandem duplication mutations), and by inhibiting FLT3 activity, it reduces the proliferation and survival of leukemia cells (1). Sorafenib has certain adverse reactions on the digestive system, skin system, endocrine system, cardiovascular system, and nervous system (6).The most frequent treatment-emergent adverse events in the sorafenib were hand–foot skin reaction (76.3%), diarrhoea (68.6%), alopecia (67.1%), and rash or desquamation (50.2%) (7).

Rhabdomyolysis is a clinical emergency syndrome characterized by acute injury or necrosis of skeletal muscle cells, involving the destruction of skeletal muscle cell membrane integrity and the release of intracellular toxic substances into the bloodstream, leading to complex metabolic disorders and organ dysfunction, and even endangering life (8, 9). The manifestations of rhabdomyolysis may range from asymptomatic to common clinical features, including acute muscle weakness, pain/tenderness, and swelling in the limbs, and dark urine (tea-colored).Rhabdomyolysis is typically caused by direct trauma; other potential etiologies include medications, toxins, infections, muscle ischemia, electrolyte and metabolic disturbances, inherited disorders, exertional stress or prolonged immobilization, and temperature-induced conditions. The diagnostic gold standard for rhabdomyolysis is laboratory measurement of plasma creatine kinase (CK) concentration, with a level exceeding five times the upper limit of the normal reference range (i.e., 1,000 IU/L) commonly used as the diagnostic threshold (10).

Rhabdomyolysis is classified as a rare adverse reaction, with only a few case reports documented over the past 15 years.Here, we report a case of rhabdomyolysis induced by sorafenib in a patient, co-occurring with hypothyroidism and hypokalemia. Laboratory findings revealed a plasma creatine kinase (CK) concentration exceeding five times the upper limit of the normal reference range.

## 2 Case report

A 31-year-old female patient was admitted to the hematology ward of our hospital on December 27, 2024, due to “bilateral lower limb weakness accompanied by intermittent numbness.” The patient has a medical history of acute myeloid leukemia and is in a post-allogeneic hematopoietic stem cell transplantation state. Prior to admission, her medications included cyclosporine soft capsules and sorafenib tablets. During the oral administration of sorafenib tablets, she experienced anorexia, foot blisters, and watery diarrhea occurring 2–3 times per night. Admission diagnosis: fatigue of undetermined etiology.The physical examination findings on admission were as follows: height, 153 cm; body weight, 45 kg; body mass index, 19.2 kg/m2; blood pressure, 115/75 mmHg; pulse, 89 beats/min; body temperature, 36.3 °C.Neurological examination demonstrated grade 4 power in the lower extremities and grade 5 power in the upper extremities, with normal muscle tone throughout.Based on the patient’s post-allogeneic hematopoietic stem cell transplantation (allo-HSCT) status, the following medications were administered during hospitalization: cyclosporine soft capsules to prevent graft-versus-host disease (GVHD), sorafenib tosylate tablets to prevent relapse of AML, and entecavir dispersible tablets to prevent hepatitis B virus reactivation.

On December 28, 2024 (Day 2), her bilateral lower limb weakness worsened, and she developed weakness in both upper limbs, making it impossible for her to sit up. She was then transferred to the neurology department for further treatment.

On December 29, 2024 (Day 3), there was no significant improvement in her limb weakness, and her mental state was poor. Laboratory tests revealed lactate dehydrogenase (LDH) 405 U/L, α-hydroxybutyrate dehydrogenase (HBDH) 243 U/L, creatine kinase (CK) 8837 U/L, cardiac creatine kinase (CK-MB) 98 U/L(**Table 1**); serum potassium (K) 2.01 mmol/L, serum calcium (Ca) 1.94 mmol/L, serum magnesium (Mg) 0.37 mmol/L (**Table 2**);thyroid function tests showed thyroxine (T4) 0.74 µg/dL, free T4 (FT4) 0.29 ng/dL, triiodothyronine (T3) < 0.20 ng/mL, free T3 (FT3) 0.97 pg/mL, thyroid-stimulating hormone (TSH) 54.750 mIU/L (**Table 3**), thyroid receptor antibodies (TRAb) 19.600 IU/L, anti-thyroglobulin antibodies (Anti-TG) > 4000.00 IU/mL, anti-thyroid peroxidase (Anti-TPO) > 600.00 IU/mL. Glomerular filtration rate (CKD-EPI equation) 120.74 ml/min,creatinine 54 *μ*mol/L.Renal function was within the normal range.Magnetic Resonance Imaging(MRI)findings suggested possible inflammatory myopathy in the bilateral thigh muscles and external obturator muscles. On the same day, oral potassium chloride solution, calcium carbonate D3 tablets, and potassium magnesium aspartate tablets were added, along with intravenous infusions of potassium chloride and compound sodium phosphate.

**Table 1:** Laboratory results of the patient’s cardiac enzyme profile and myoglobin levels.

Date	Creatine kinase(40-200U/L)	Lactate dehydrogenase(120-250U/L)	α-hydroxybutyrate dehydrogenase(90-180U/L)	Cardiac creatine kinase(0-24U/L)	Myoglobin (25-58ng/mL)
2023/11/28	46.00	204.00	148.00	18.00	–
2024/12/29	8837.00	405.00	243.00	98.00	–
2024/12/29	10588.00	394.00	249.00	88.00	–
2024/12/30	16066.00	496.00	249.00	114.00	2973.00
2024/12/31	21287.00	595.00	298.00	140.00	1419.00
2025/1/2	10468.00	379.00	235.00	115.00	876.00
2025/1/4	6982.00	443.00	257.00	88.00	902.00
2025/1/6	3657.00	326.00	198.00	52.00	514.00
2025/1/8	2025.00	282.00	188.00	38.00	352.00
2025/1/16	882.00	264.00	196.00	41.00	164.00
2025/2/17	120.00	188.00	133.00	86.00	–

The normal reference range of the laboratory test results is shown in parentheses.

**Table 2:** Examination results of patient’s electrolytes.

Date	K(3.5-5.3mmol/L)	Ca(2.11-2.52mmol/L)	Mg(0.75-1.02mmol/L)
2023/11/28	3.82	2.47	1.02
2024/12/29	2.01	1.94	0.37
2024/12/29	2.22	1.87	0.42
2024/12/29	2.43	1.84	0.42
2024/12/30	2.81	1.91	0.41
2024/12/30	3.97	1.84	0.42
2024/12/31	4.65	2.01	0.51
2025/1/2	4.04	2.40	0.58
2025/1/4	4.42	2.41	0.76
2025/1/6	4.15	2.22	0.80
2025/1/8	4.09	2.38	0.73
2025/1/16	4.21	2.46	0.82
2025/2/17	4.21	2.32	0.83

The normal reference range of the laboratory test results is shown in parentheses.

**Table 3:** Patient’s thyroid function test results.

Date	T4 (4.87-11.72ug/dl)	FT4 (0.70-1.48ng/dl)	T3 (0.64-1.52ng/ml)	FT3 (1.58-3.91pg/ml)	TSH (0.35-4.94mIU/L)
2024/12/29	0.74	0.29	<0.2	0.97	54.75
2025/1/4	0.81	0.34	<0.2	1.01	66.472

The normal reference range of the laboratory test results is shown in parentheses.

On December 30, 2024 (Day 4), the patient’s limb weakness improved slightly, but she had stiff hand muscles and poor mental state. Laboratory tests showed myoglobin measurement at 2973.00 ng/mL, with continued deterioration of myocardial enzyme spectrum indicators, while serum potassium gradually returned to normal. After consultation with a clinical pharmacist, it was suspected that sorafenib induced the patient’s rhabdomyolysis. The recommendation was to temporarily discontinue sorafenib, add calcitriol, and continue calcium carbonate D3 supplementation and thyroid function correction. Oral levothyroxine was also initiated on the same day.In addition, the patient received intravenous infusion of potassium chloride injection as an active treatment to correct hypokalemia.

On December 31, 2024 (Day 5), sorafenib was discontinued, and the patient continued to receive oral potassium chloride, calcitriol, calcium carbonate D3, and levothyroxine. From the 5th to the 12th day of hospitalization, the patient received a daily infusion of 1500 ml to 3000 ml of sodium chloride injection to accelerate the renal excretion of sorafenib.

By Day 4 of hospitalization, the patient’s potassium levels had returned to normal, and electrolyte imbalances showed gradual improvement. Following the cessation of sorafenib, the patient’s limb weakness progressively ameliorated, with satisfactory mental state and sleep quality.

**Figure 1:**
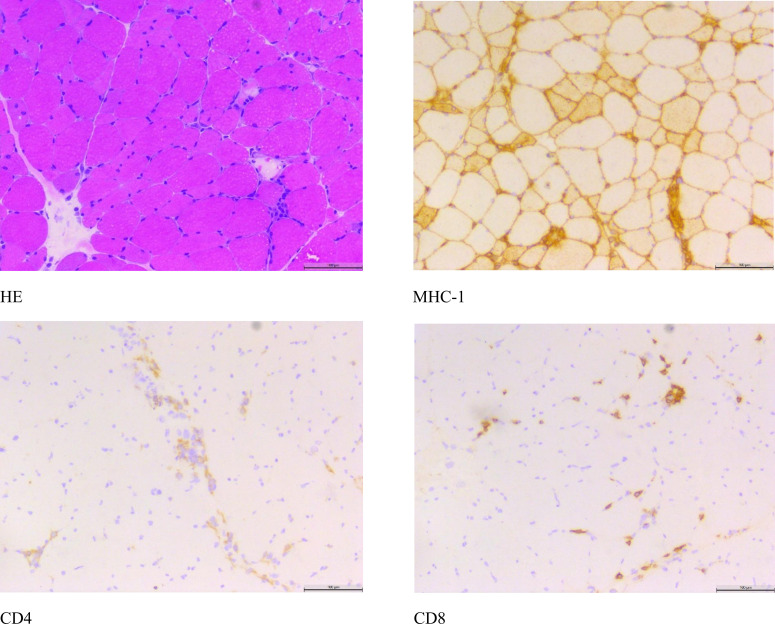
Nerve and muscle biopsy histological staining.

On January 9, 2025 (Day 12), the patient’s condition was stable with a satisfactory general status, leading to subsequent discharge. The discharge diagnosis was rhabdomyolysis. Laboratory studies revealed a creatine kinase level of 2,025 U/L and a potassium (K+) level of 4.09 mmol/L.On January 13, 2025,histochemical staining of the neuromuscular biopsy specimen indicated histopathological changes consistent with rhabdomyolysis, with differential considerations including immune-mediated necrotizing myopathy (**Figure 1**).

The patient was followed in the outpatient clinic after discharge, showing improvement in lower extremity muscle weakness.On February 17, 2025, follow-up laboratory tests showed a creatine kinase level of 120 U/L and a potassium (K+) level of 4.21 mmol/L.

## 3 Discussion

Upon admission, the patient was afebrile and had a white blood cell count within normal limits. No clinical signs suggestive of infection were observed, and the patient denied any history of strenuous physical activity. The patient’s pre-admission medication regimen was simple, primarily consisting of Cyclosporine Soft Capsules and Sorafenib Tablets.A review of the patient’s laboratory results in November 2023 indicated a potassium (K+) level of 3.82 mmol/L and a creatine kinase (CK) level of 46 U/L, both falling within their respective normal reference ranges.

Furthermore, direct trauma, a common etiology of rhabdomyolysis, was ruled out.It is hypothesized that the patient’s recent onset of rhabdomyolysis and hypokalemia are likely adverse drug reactions secondary to the initiation of oral Sorafenib therapy in March 2024. Following the discontinuation of Sorafenib, the patient’s creatine kinase levels demonstrated a gradual decline.During the hospitalization, the patient received potassium chloride supplementation, resulting in a progressive normalization of serum potassium levels. Notably, the peak CK level during admission reached 21,287 U/L, exceeding the upper limit of the normal reference range by more than tenfold.Histopathological examination of a neuromuscular biopsy revealed scattered necrotic, degenerating, and regenerating muscle fibers on Hematoxylin and Eosin (H&E) staining. Immunohistochemical studies showed partial sarcolemmal positivity for MHC-I, with scattered CD8-positive and CD4-positive cells. These histopathological findings are consistent with a diagnosis of rhabdomyolysis and immune-mediated necrotizing myopathy.

Rhabdomyolysis induced by sorafenib therapy is exceedingly rare. In 2013, a case of rhabdomyolysis associated with sorafenib treatment for advanced hepatocellular carcinoma was reported (11), followed by another case in 2020 involving a patient with multifocal hepatocellular carcinoma undergoing sorafenib therapy (12). Unlike this case, neither of the two reported cases was accompanied by hypokalemia or hypothyroidism.This suggests that sorafenib-induced rhabdomyolysis may not necessarily alter serum potassium levels or induce hypothyroidism. The underlying mechanism of sorafenib-related rhabdomyolysis remains incompletely understood but may involve inhibition of mitochondrial complex activity and disruption of glucose and nucleotide uptake, ultimately impairing energy metabolism in skeletal muscle cells (13).

In this case, the patient’s rhabdomyolysis was accompanied by hypokalemia, which may be attributed to chronic anorexia and diarrhea leading to potassium depletion and inadequate potassium intake. Studies have reported that hypokalemia itself can induce rhabdomyolysis (14, 15). Specifically, hypokalemia further inhibits Na+-K+-ATPase activity, compromises membrane potential stability, and contributes to muscle weakness (16). Any process that impairs ATP production in skeletal muscle or creates a state where energy demand exceeds available ATP supply may precipitate rhabdomyolysis (9). Hypokalemia increases the risk of rhabdomyolysis by disrupting muscular energy metabolism and impairing blood flow regulation (17). Thus, we propose that sorafenib-associated rhabdomyolysis in this patient likely resulted from both direct drug effects and indirect mechanisms mediated by hypokalemia, acting through combined pathways.

During hospitalization, the patient was diagnosed with hypothyroidism. Due to the lack of pre-admission thyroid function data, it could not be determined whether hypothyroidism was already present before admission. During the hospital stay, the patient’s TSH levels exceeded the normal range and showed a continuous upward trend, indicating progression of the hypothyroid state. Although rhabdomyolysis occurred concomitantly with hypothyroidism, its severity did not appear to progress in parallel with worsening thyroid function indices during the clinical course. While severe hypothyroidism alone can precipitate rhabdomyolysis, the present episode likely has a multifactorial etiology. In this context, the coexistence of hypothyroidism and hypokalemia in the patient may have served as synergistic risk factors for the development of rhabdomyolysis.

**Figure 2:**
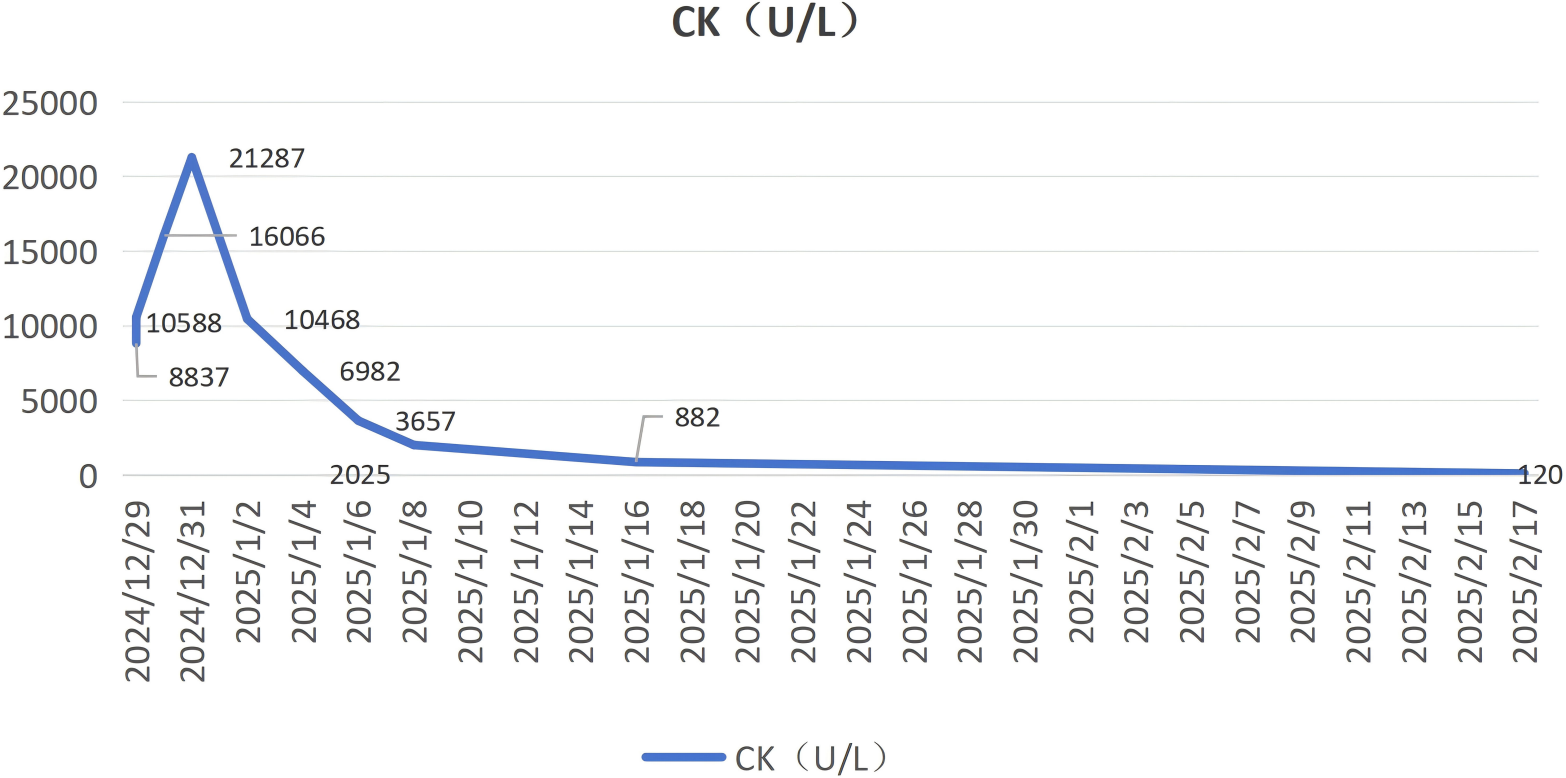
Trends in Creatine Kinase (CK) levels.

Meanwhile, we utilized the Naranjo Adverse Drug Reaction (ADR) Probability Scale to standardize the assessment of causality between sorafenib and rhabdomyolysis. The total Naranjo score was 5, indicating a “Probable” causal relationship between sorafenib and rhabdomyolysis. This conclusion is primarily supported by the following key clinical observations: First, sorafenib-induced rhabdomyolysis has been previously documented in the literature. Second, a clear temporal association was observed—the ADR emerged following sorafenib administration and showed significant improvement after its discontinuation, consistent with the typical time course of a drug-induced reaction. Although the patient also presented with hypokalemia, serum potassium levels were corrected by the fourth day of hospitalization via oral and intravenous supplementation. Despite this, the creatine kinase (CK) level continued to rise, peaking on the fifth day of hospitalization. However, after discontinuation of sorafenib, the CK level decreased markedly from 21,287 U/L to 10,468 U/L (**Figure 2**). Additionally, histochemical staining of the neuromuscular biopsy suggested rhabdomyolysis in the context of immune-mediated necrotizing myopathy.Finally, although we systematically excluded major causes of rhabdomyolysis such as direct trauma, infection, toxins, and other medications, the possibility of hypothyroidism prior to the patient’s hospital admission cannot be ruled out.

## 4 Conclusions

In clinical practice, serious attention should be paid to the adverse reactions that may be induced by sorafenib, particularly its potential to cause rhabdomyolysis—a rare yet life-threatening adverse event. A CK level exceeding 15,000 U/L indicates severe rhabdomyolysis and increases the risk of renal impairment and the need for dialysis therapy (10). Therefore, it is recommended that patients undergoing sorafenib treatment receive routine monitoring and evaluation of CK and electrolytes, with vigilance against the potentially serious adverse effects of rhabdomyolysis.

## Data Availability

The original contributions presented in the study are included in the article/supplementary material. Further inquiries can be directed to the corresponding author.
